# *catena*-Poly[cadmium(II)-μ-benzene-1,2-diamine-κ^2^ *N*:*N*′-di-μ-chlorido]

**DOI:** 10.1107/S1600536808027980

**Published:** 2008-09-06

**Authors:** Wen-Xian Liang, Zhi-Rong Qu

**Affiliations:** a Ordered Matter Science Research Center, College of Chemistry and Chemical Engineering, Southeast University, Nanjing 210096, People’s Republic of China

## Abstract

The title compound, [CdCl_2_(C_6_H_8_N_2_)]_*n*_, is a coordination polymer prepared by the hydro­thermal reaction of cadmium chloride and *o*-diamino­benzene. The cadmium cation, located on an inversion center, is octa­hedrally coordinated by four Cl atoms at equatorial sites and two N atoms from two ligands at the axial sites. Cd atoms are bridged by Cl atoms, forming extended chains parallel to [010]. Neighboring chains are connected by N—H⋯Cl hydrogen bonds.

## Related literature

For related literature, see: Choi *et al.* (1999 ▶); Spingler *et al.* (2001 ▶); Fu & Zhao (2007 ▶).
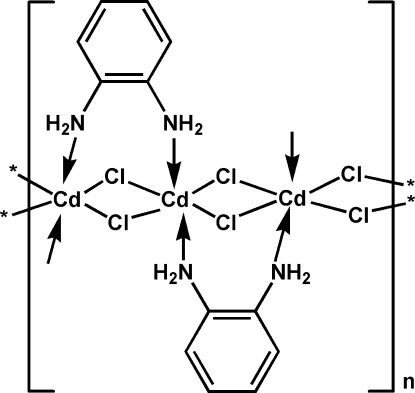


## Experimental

### 

#### Crystal data

[CdCl_2_(C_6_H_8_N_2_)]
*M* *_r_* = 291.44
Monoclinic, 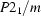

*a* = 6.1235 (6) Å
*b* = 7.5473 (5) Å
*c* = 10.1081 (6) Å
β = 105.23 (10)°
*V* = 450.75 (6) Å^3^
*Z* = 2
Mo *K*α radiation
μ = 2.95 mm^−1^
*T* = 293 (2) K
0.18 × 0.15 × 0.14 mm

#### Data collection

Rigaku SCXmini diffractometer
Absorption correction: multi-scan (*CrystalClear*; Rigaku, 2005 ▶) *T*_min_ = 0.595, *T*_max_ = 0.660
4700 measured reflections
1109 independent reflections
1020 reflections with *I* > 2σ(*I*)
*R*_int_ = 0.029

#### Refinement

*R*[*F* ^2^ > 2σ(*F* ^2^)] = 0.018
*wR*(*F* ^2^) = 0.040
*S* = 1.22
1109 reflections
56 parameters
H-atom parameters constrained
Δρ_max_ = 0.25 e Å^−3^
Δρ_min_ = −0.35 e Å^−3^

### 

Data collection: *CrystalClear* (Rigaku, 2005 ▶); cell refinement: *CrystalClear*; data reduction: *CrystalClear*; program(s) used to solve structure: *SHELXS97* (Sheldrick, 2008 ▶); program(s) used to refine structure: *SHELXL97* (Sheldrick, 2008 ▶); molecular graphics: *SHELXTL/PC* (Sheldrick, 2008 ▶); software used to prepare material for publication: *SHELXL97* and *PRPKAPPA* (Ferguson, 1999 ▶).

## Supplementary Material

Crystal structure: contains datablocks global, I. DOI: 10.1107/S1600536808027980/bx2174sup1.cif

Structure factors: contains datablocks I. DOI: 10.1107/S1600536808027980/bx2174Isup2.hkl

Additional supplementary materials:  crystallographic information; 3D view; checkCIF report

## Figures and Tables

**Table 1:** Hydrogen-bond geometry (Å, °)

*D*—H⋯*A*	*D*—H	H⋯*A*	*D*⋯*A*	*D*—H⋯*A*
N1—H1*B*⋯Cl1^i^	0.89	2.51	3.3960 (18)	171

Symmetry code: (i) 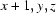.

**Fig. 1.**
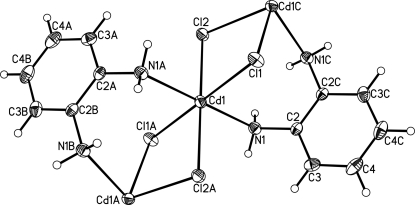
A partial packing diagram of the title compound, with the displacement ellipsoids were drawn at the 30% probability level. [Symmetry codes: (A) 1-x, -y, 1-z; (B) 1-x, -12+y, 1-z; (C) +x, 1/2-y, +z.]

**Fig. 2.**
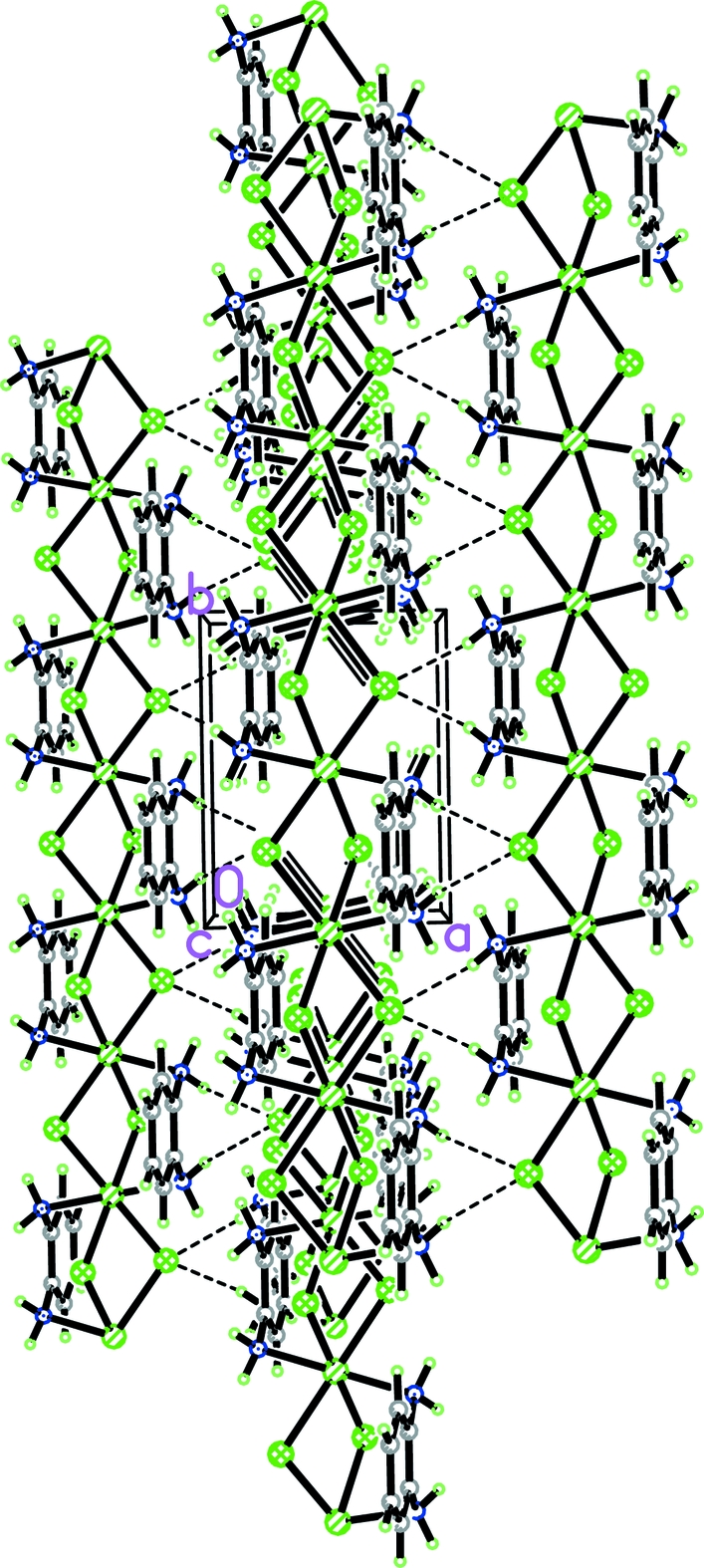
Part of the structure of (I), showing two-dimensional extended polymeric network. Dotted lines show intermolecular hydrogen bonding.

## supplementary crystallographic information

### Comment

Coordination frameworks have received much attention over the past decade due to their potential applications. Scientists have dedicated much attention to coordination compounds which constructed by ligands with diamino coordination sites (Choi *et al.*, 1999; Fu *et al.*, 2007), since *cis*-diamminedichloroplatium(II) received Food and Drug Administration's approval in 1979 for use as an anticancer drug (Spingler *et al.*, 2001). The title compound, [CdCl_2_(C_6_H_8_N_2_)]_n_, is a coordination polymer prepared by the hydrothermal reaction of cadmium chloride and *o*-diaminobenzene.The Cd cation is located on the inversion center and octahedrally coordinated by four Cl atoms at equatorial sites and two N atoms from two ligands at the axial sites. Cd cations are bridged by Cl atoms to form a one-dimensional extended chain. The neighboring chains are binded by N—H···Cl hydrogen bonds.(Table 1) to form a two-dimensional network (Fig. 2).

### Experimental

A mixture of CdCl_2_ (0.0366 g, 0.2 mmol) and benzene-1,2-diamine (0.0216 g, 0.2 mmol) in H_2_O (4 ml) was heated in Pyrex tube at 100°C for two days. After slowly cooling down to room temperature over a period of 10 h, colorless crystals of the title compound suitable for diffraction were isolated.

### Refinement

Positional parameters of all the H atoms were calculated geometrically and were allowed to ride on the C, N atoms to which they are bonded, with C—H = 0.93 Å, N—H = 0.90 Å and with *U*_iso_(H) = 1.2 *U*_eq_(C).

### Crystal data

**Table:**

[CdCl_2_(C_6_H_8_N_2_)]	*F*(000) = 280
*M**_r_* = 291.44	*D*_x_ = 2.147 Mg m^−^^3^
Monoclinic, *P*2_1_/*m*	Mo *K*α radiation, λ = 0.71073 Å
Hall symbol: -P 2yb	Cell parameters from 4460 reflections
*a* = 6.1235 (6) Å	θ = 3.1–27.5°
*b* = 7.5473 (5) Å	µ = 2.95 mm^−^^1^
*c* = 10.1081 (6) Å	*T* = 293 K
β = 105.230 (1)°	Prism, colourless
*V* = 450.75 (6) Å^3^	0.18 × 0.15 × 0.14 mm
*Z* = 2	

### Data collection

**Table:**

Rigaku SCXmini diffractometer	1109 independent reflections
Radiation source: fine-focus sealed tube	1020 reflections with *I* > 2σ(*I*)
graphite	*R*_int_ = 0.029
Detector resolution: 13.6612 pixels mm^-1^	θ_max_ = 27.5°, θ_min_ = 3.4°
CCD profile fitting scans	*h* = −7→7
Absorption correction: multi-scan (CrystalClear; Rigaku, 2005)	*k* = −9→9
*T*_min_ = 0.595, *T*_max_ = 0.660	*l* = −13→13
4700 measured reflections	

### Refinement

**Table:**

Refinement on *F*^2^	Secondary atom site location: difference Fourier map
Least-squares matrix: full	Hydrogen site location: inferred from neighbouring sites
*R*[*F*^2^ > 2σ(*F*^2^)] = 0.018	H-atom parameters constrained
*wR*(*F*^2^) = 0.040	*w* = 1/[σ^2^(*F*_o_^2^) + (0.0114*P*)^2^ + 0.0467*P*] where *P* = (*F*_o_^2^ + 2*F*_c_^2^)/3
*S* = 1.22	(Δ/σ)_max_ < 0.001
1109 reflections	Δρ_max_ = 0.25 e Å^−^^3^
56 parameters	Δρ_min_ = −0.35 e Å^−^^3^
0 restraints	Extinction correction: SHELXL97 (Sheldrick, 1997), Fc^*^=kFc[1+0.001xFc^2^λ^3^/sin(2θ)]^-1/4^
Primary atom site location: structure-invariant direct methods	Extinction coefficient: 0.115 (2)

### Special details

**Table:**

Geometry. All e.s.d.'s (except the e.s.d. in the dihedral angle between two l.s. planes) are estimated using the full covariance matrix. The cell e.s.d.'s are taken into account individually in the estimation of e.s.d.'s in distances, angles and torsion angles; correlations between e.s.d.'s in cell parameters are only used when they are defined by crystal symmetry. An approximate (isotropic) treatment of cell e.s.d.'s is used for estimating e.s.d.'s involving l.s. planes.
Refinement. Refinement of *F*^2^ against ALL reflections. The weighted *R*-factor *wR* and goodness of fit *S* are based on *F*^2^, conventional *R*-factors *R* are based on *F*, with *F* set to zero for negative *F*^2^. The threshold expression of *F*^2^ > σ(*F*^2^) is used only for calculating *R*-factors(gt) *etc*. and is not relevant to the choice of reflections for refinement. *R*-factors based on *F*^2^ are statistically about twice as large as those based on *F*, and *R*- factors based on ALL data will be even larger.

### Fractional atomic coordinates and isotropic or equivalent isotropic displacement parameters (Å^2^)

**Table:**

	*x*	*y*	*z*	*U*_iso_*/*U*_eq_	
Cd1	0.5000	0.0000	0.5000	0.02699 (11)	
Cl1	0.26524 (11)	0.2500	0.58281 (8)	0.03186 (18)	
Cl2	0.62145 (13)	0.2500	0.35295 (7)	0.03336 (19)	
N1	0.8292 (3)	0.0596 (2)	0.67983 (17)	0.0290 (4)	
H1A	0.8687	−0.0546	0.7042	0.043*	
H1B	0.9341	0.1080	0.6439	0.043*	
C2	0.7996 (3)	0.1574 (2)	0.79569 (18)	0.0249 (4)	
C3	0.7561 (3)	0.0677 (3)	0.9058 (2)	0.0348 (5)	
H3	0.7530	−0.0555	0.9056	0.042*	
C4	0.7174 (3)	0.1588 (3)	1.0153 (2)	0.0411 (5)	
H4	0.6913	0.0971	1.0893	0.049*	

### Atomic displacement parameters (Å^2^)

**Table:**

	*U*^11^	*U*^22^	*U*^33^	*U*^12^	*U*^13^	*U*^23^
Cd1	0.03100 (15)	0.02342 (14)	0.02722 (15)	−0.00010 (7)	0.00882 (10)	−0.00260 (7)
Cl1	0.0293 (4)	0.0263 (4)	0.0466 (4)	0.000	0.0218 (3)	0.000
Cl2	0.0499 (4)	0.0270 (4)	0.0289 (4)	0.000	0.0205 (3)	0.000
N1	0.0290 (9)	0.0270 (8)	0.0324 (9)	−0.0003 (7)	0.0108 (8)	−0.0011 (7)
C2	0.0180 (8)	0.0319 (10)	0.0234 (9)	−0.0015 (7)	0.0031 (7)	−0.0008 (8)
C3	0.0284 (10)	0.0391 (12)	0.0348 (11)	−0.0017 (9)	0.0045 (9)	0.0089 (10)
C4	0.0308 (11)	0.0662 (15)	0.0271 (10)	−0.0034 (10)	0.0093 (9)	0.0071 (10)

### Geometric parameters (Å, °)

**Table:**

Cd1—N1^i^	2.3758 (17)	N1—H1A	0.9113
Cd1—N1	2.3758 (17)	N1—H1B	0.8946
Cd1—Cl2	2.6274 (5)	C2—C3	1.387 (3)
Cd1—Cl2^i^	2.6274 (5)	C2—C2^iii^	1.398 (4)
Cd1—Cl1^i^	2.6381 (5)	C3—C4	1.375 (3)
Cd1—Cl1	2.6381 (5)	C3—H3	0.9300
Cl1—Cd1^ii^	2.6381 (5)	C4—C4^iii^	1.377 (5)
Cl2—Cd1^ii^	2.6274 (5)	C4—H4	0.9300
N1—C2	1.436 (2)		
			
N1^i^—Cd1—N1	180.00 (7)	Cd1—Cl2—Cd1^ii^	91.80 (2)
N1^i^—Cd1—Cl2	90.71 (4)	C2—N1—Cd1	117.26 (11)
N1—Cd1—Cl2	89.29 (4)	C2—N1—H1A	110.3
N1^i^—Cd1—Cl2^i^	89.29 (4)	Cd1—N1—H1A	98.0
N1—Cd1—Cl2^i^	90.71 (4)	C2—N1—H1B	112.2
Cl2—Cd1—Cl2^i^	180.0	Cd1—N1—H1B	108.8
N1^i^—Cd1—Cl1^i^	92.61 (4)	H1A—N1—H1B	109.2
N1—Cd1—Cl1^i^	87.39 (4)	C3—C2—C2^iii^	119.20 (13)
Cl2—Cd1—Cl1^i^	94.319 (17)	C3—C2—N1	119.72 (18)
Cl2^i^—Cd1—Cl1^i^	85.682 (17)	C2^iii^—C2—N1	120.96 (10)
N1^i^—Cd1—Cl1	87.39 (4)	C4—C3—C2	120.8 (2)
N1—Cd1—Cl1	92.61 (4)	C4—C3—H3	119.6
Cl2—Cd1—Cl1	85.682 (17)	C2—C3—H3	119.6
Cl2^i^—Cd1—Cl1	94.318 (17)	C3—C4—C4^iii^	119.98 (13)
Cl1^i^—Cd1—Cl1	180.00 (3)	C3—C4—H4	120.0
Cd1^ii^—Cl1—Cd1	91.32 (2)	C4^iii^—C4—H4	120.0

Symmetry codes: (i) −*x*+1, −*y*, −*z*+1; (ii) −*x*+1, *y*+1/2, −*z*+1; (iii) *x*, −*y*+1/2, *z*.

### Hydrogen-bond geometry (Å, °)

**Table:**

*D*—H···*A*	*D*—H	H···*A*	*D*···*A*	*D*—H···*A*
N1—H1B···Cl1^iv^	0.89	2.51	3.3960 (18)	171.

Symmetry codes: (iv) *x*+1, *y*, *z*.
